# Editorial: Plant Phenotyping and Phenomics for Plant Breeding

**DOI:** 10.3389/fpls.2017.02181

**Published:** 2017-12-22

**Authors:** Gustavo A. Lobos, Anyela V. Camargo, Alejandro del Pozo, Jose L. Araus, Rodomiro Ortiz, John H. Doonan

**Affiliations:** ^1^PIEI Adaptación de la Agricultura al Cambio Climático, Facultad de Ciencias Agrarias, Plant Breeding and Phenomic Center, Universidad de Talca, Talca, Chile; ^2^The John Bingham Laboratory, Genetics and Breeding, National Institute of Agricultural Botany, Cambridge, United Kingdom; ^3^Plant Physiology Section, University of Barcelona, Barcelona, Spain; ^4^Department of Plant Breeding, Swedish University of Agricultural Sciences, Alnarp, Sweden; ^5^National Plant Phenomics Centre, Institute of Biological, Environmental and Rural Sciences, Aberystwyth University, Aberystwyth, United Kingdom

**Keywords:** Latin America, high-throughput phenotyping, forward phenomics, reverse phenomics, software development

## Introduction

A major challenge for food production in the coming decades is to meet the food demands of a growing population (Beddington, 2010). The difficulty of expanding agricultural land, along with the effect of climate change and the increase in world population are the current societal changes that make necessary to accelerate research to improve yield-potential and adaptation to stressful environments (Lobos et al., 2014; Camargo and Lobos). Increasing yields will require implementing novel approaches in gene discovery and plant breeding that will significantly increase both production per unit of land area and resource use efficiency (Parry and Hawkesford, 2010; Tanger et al., 2017). A critical component for accelerating the development of new and improved cultivars is the rapid and precise phenotypic assessment of thousands of breeding lines, clones or populations over time (Fu, 2015) and under diverse environments. The only reasonable way to satisfy all these demands is through acquisition of high-dimensional phenotypic data (high-throughput phenotyping) or “phenomics” (Houle et al., 2010). This approach may predict complex characters that are relevant for plant selection (forward phenomics), and will also provide explanations as to why given genotypes stands out in a specific environment (reverse phenomics) (Camargo and Lobos).

Phenotype can be defined as the characteristics of an organism resulting from the interaction between genotype, environment and crop management. Phenomics involves the gathering of appropriate phenotypic data at multiple levels of organization, to progress toward a more complete characterization of phenotypic space generated by a particular genome or set of genomes (Dhondt et al., 2013). Thus, plant phenotyping can operate at different levels of resolution and dimensionality, from the molecular to the whole plant, and in different environments, from controlled to field conditions. Although, each level focuses on particular traits, the ultimate goal is to integrate knowledge from the bottom up to produce cultivars with higher performance. In that regard, the use of plant phenotyping methods as part of breeding programs has become a powerful research tool to help breeders generate cultivars more adaptable to diverse challenging environmental scenarios (Camargo and Lobos).

This Research Topic (RT) issue is based on contributions from invited speakers to the First Latin-American Conference on Plant Phenotyping and Phenomics for Plant Breeding carried out during 2015 at Universidad de Talca (Talca, Chile), and from other scientists who are currently researching on phenomics and plant breeding. Thus, the categories and scope are diverse (review, perspective, and original research) and address different objectives through various levels of resolution and dimensionality. Interestingly, even though most of the phenotyping and phenomics for plant breeding research have been developed for model plants and cereals (Lobos and Hancock; Camargo and Lobos), this RT highlights the feasibility of implement these approaches on breeding programs targeted to other crop species.

## Characterization of the plant: from the gene to population responses by remote sensing

Many of the articles comment on knowledge gained from model plants that was applied to crops. For example, Liu et al. proposed a pathway model for trichome development in cucumber and compared it with the model from *Arabidopsis thaliana*. Yu et al. identified *VaERD15* as a transcription factor gene associated with cold-tolerance in Chinese wild *Vitis amurensis*, and the expression levels increased after low-temperature treatment, enhancing cold tolerance. Cao et al. analyzed gene expression on transgenic material to unveil the functional roles of four expressed *FT*-like genes in tomato. These authors also demonstrated the functional differentiation between *FT*-like genes in controlling flowering through overexpression in *A. thaliana* and VIGS-mediated knocking down in tomato. Awlia et al. were able to show further that phenotyping multiple quantitative traits in one experimental setup can provide new insights into the dynamics of plant responses to stress, suggesting the use of forward genetics studies to identify genes underlying early responses to stress.

The small grain cereals are very well represented in this RT issue. For example, Wu et al. found that overexpressing *OsDof12* in rice could lead to reduced plant height, erected leaf, shortened leaf blade, and smaller panicle resulted from decreased number of primary and secondary branches. Barber et al. performed 1-day transfers of pot-grown wheat to replicated controlled environments, providing strong evidence that the key phases susceptible to heat stress at booting and anthesis in wheat are discrete and that genotypes vary with regards to the most susceptible growth stage; at anthesis, the north European allele *Rht-D1b* was related with higher tolerance to heat stress. Adriani et al. analyzed the effect of different quantitative trait loci (QTL) and their interaction with growing conditions on panicle size and number in rice. Their results showed that grain production was enhanced by *qTSN* only under shading conditions, where panicle number was not affected while photosynthesis and starch storage in internodes were enhanced. Similarly, Camargo et al. established the value of systematically phenotyping genetically unstructured populations to reveal the genetic architecture underlying morphological variation in commercial wheat, QTL with phenological characters such as heading, and the onset of flag leaf senescence, as well as morphological traits such as stem height. In order to have consistency between stress conditions and seasons, del Pozo et al. highlighted the importance of defining and deeply characterizing the target environment before determining the set of phenotyping traits for selection.

Integration of molecular analyses with whole plant phenotyping deepens our understanding of responses to environmental variables as demonstrated by Sanchez-Bragado et al. who proposed the combination of δ^15^N and N content as an affordable tool to phenotype the relative contribution of different plant parts to the grain N in wheat under contrasting water and nitrogen conditions. Similarly, as selection criteria in breeding N-efficient rice cultivars, Wu et al. suggested that promoting pre-heading growth could increase total nitrogen uptake at maturity, while high biomass accumulation during the grain filling period and large panicles are important for higher nitrogen use efficiency for grain production. Nigro et al. also suggested the use of glutamine synthetase activity and expression as a candidate proxy to select genotypes having high grain protein content in wheat. Fisher et al. found that at early stages of drought stress, metabolic profiling could be used as an efficient tool to discriminate among tolerant or susceptible genotypes of the model plant *Brachypodium distachyon*. Medina et al. concluded for wheat that the combination of phenotyping and gene expression analysis is a useful approach to identify phenotype-genotype relationships and their behavior in response to different environments, which mostly follows from the combination of water regimes and CO_2_ levels during vegetative stages.

Kumar et al. used molecular, phenotypic, and geographical diversity to develop a compact composite core collection in the oilseed crop safflower (*Carthamus tinctorius* L.) that will facilitate the identification of genetic determinants of trait variability. Mora et al. emphasized that large number of spurious QTL could be detected when the genetic covariance matrix is ignored in the mixed model analysis, increasing the rate of false-positives. In strawberry breeding programs, Hancock et al. proved that much of the cost associated with DNA marker discovery for marker-aided breeding (MAB) can be eliminated if a diverse, segregating population is generated, genotyped, and made available to the global breeding community. Tan et al. proposed the use of Digital Gen Expression (DGE) as an efficient tool to find differences in transcriptional responses of different tissues/organs of castor plants (*Ricinus communis* L.) subjected to stress, but also to understand molecular mechanisms associated to sex variation. Among approaches to improve yield potential, delayed leaf senescence or stay-green attributes were also addressed in this RT (Balakrishnan et al., 2016; Camargo et al.; Fisher et al.; Wu et al.; Yang et al.; del Pozo et al.).

## Novel methodological approaches and software development

This issue also considers methodological approaches to characterize and classify cells and to quantify fluorescence at sub-cellular level (Hall et al.). Likewise, using a wheat panel of elite cultivars and non-adapted genetic resources growing under different adverse environments, Tattaris et al. compared on-ground proximal assessments of canopy temperature (CT) and NDVI with data collected from unmanned aerial vehicles (UAV) and satellite; considering statistical analyses, cost and the feasibility of performing measurements of a high number of genotypes at any moment, the authors recommend the use of UAV for plant breeding purposes. Vergara-Díaz et al. highlighted the advantages of using spectral reflectance indices (SRI) derived from Red-Green-Blue (RGB) digital images as a low-cost tool for prediction of several traits (e.g., grain yield, leaf N concentration and the ratio of carbon to nitrogen) that are highly valuable for maize breeders. Likewise, Garriga et al. proposed the use of classification methods (PLS-DA) as an efficient tool to directly identify the elite genotypes group instead to focus on the estimation of predicted trait-values.

Software developments were also highlighted in this RT. For example, Deery et al. showed the efficiency of a custom-developed airborne thermography image processing software (ChopIt), used for plot segmentation and extraction of canopy temperature from each individual plot by a non-technical user. Kaushik et al. performed a deep characterization of eggplant using the Tomato Analyzer (Rodríguez et al., 2010). Using Spectral Knowledge (SK-UTALCA; Lobos and Poblete-Echeverría, 2017), Garriga et al. performed an exploratory analysis of high-resolution spectral reflectance data, testing 255 SRI at the same time. RGB images were filtered by a retina filter (Benoit et al., 2010), which enhanced the contrast between plant and background, improving color consistency and providing spatial noise removal and luminance correction (Fisher et al.). Awlia et al. used the PlantScreen™Compact System (PSI, Czech Republic) equipped with chlorophyll fluorescence and RGB imaging, as well as with automatically weighing and watering of plants to test the response of *A. thaliana* to salinity. The open source Breedpix 0.2 software (Casadesus et al., 2007) was used by Vergara-Díaz et al. for the calculation of several RGB vegetation indices based on the different properties of color inherent in RGB images.

## Conclusions

This RT highlights the importance of a holistic characterization of the crop, from the cell to the plant level using for that current available tools for plant phenotyping. Gathering, integrating, and making inferences on these data will help breeding programs to speed up the release of cultivars tolerant to stress environments. Alongside more data, training programs should be established to guarantee the use and adoption of new technologies.

In addition to aspects associated to phenomics and breeding, the RT also discussed future challenges such as the need for multi-disciplinary research and the better and deeper characterization of the environment. The progress of forward and reverse phenomics has great potential to accelerate the improvement of yield potential and we expect to see that rapid developments will continue in this subject area, especially in Latin America.

## Author contributions

GL, AC, AdP, JA, RO, and JD co-wrote this editorial based on the various contributions to this Research Topic.

### Conflict of interest statement

The authors declare that the research was conducted in the absence of any commercial or financial relationships that could be construed as a potential conflict of interest.
